# The Challenges of Eating Well for People Living with Cystic Fibrosis: an Interview Study Exploring the Use of Mindful Eating Approaches and Behaviours to Support Optimal Nutritional Status

**DOI:** 10.1007/s12529-022-10057-x

**Published:** 2022-01-27

**Authors:** Helen Egan, Rebecca Keyte, Michail Mantzios

**Affiliations:** Faculty of Business, Law and Social Sciences, Department of Psychology, Birmingham City University, Room C332, The Curzon Building, 4 Cardigan St., Birmingham, B4 7BD UK

**Keywords:** Cystic fibrosis, Mindfulness, Eating behaviours, Mindful eating, Interventions

## Abstract

**Background:**

Nutritional status and weight are closely linked to lung function and health status in cystic fibrosis (CF). The investigation of eating behaviours has shown mindfulness practices to be useful in modifying eating behaviours, particularly with obesity; to date, no research specifically explores how these concepts may be utilised within a CF population who face specific challenges in eating behaviours.

**Method:**

Adult patients (*n* = 20, *M* = 8, age range 21–62 years) were recruited from a UK CF regional centre to take part in qualitative semi-structured interviews. Experiences of eating behaviours focusing on the use of mindful (or mindless) eating as barriers and enablers to achieving and maintaining optimal nutritional status were explored. Data were analysed using thematic analysis with a contextualist approach to understand how participants experienced eating behaviours within the context of health and weight status.

**Results:**

Participants engaged readily in discussions of eating behaviour describing active self-regulation of food eaten and calorie intake. Participants who struggled to maintain weight employed strategies to increase calorie intake such as distraction and multitasking while eating. Most participants reported no pleasure in food, describing eating as another treatment to endure. Confusion existed around how to eat healthily for CF alongside co-morbid health conditions including diabetes, cholesterol and heart disease.

**Conclusion:**

Participants were highly aware of their eating behaviours, engaging in intentional and deliberate preparations, which could be described as mindful, for making eating a more automatic or mindless activity. Modifications to usual mindful eating interventions are needed to support people with CF.

## Introduction

Cystic fibrosis (CF) is an autosomal recessive inherited disorder affecting up to 100,000 people worldwide and is the most common genetic disease in Caucasian North European populations. This disorder results in complex symptoms necessitating intense clinical management that impacts considerably on eating behaviours. CF is caused by defects in a recessive gene, the cystic fibrosis transmembrane conductance regulator (CFTR). The resulting imbalance in fluids and salts increases the production of thick, sticky mucus and impairs the functioning of the respiratory tract, inhibiting the clearance of microorganisms leading to recurrent infections. Developments in treatments in the last 20 years have led to increases in life expectancy for people with CF in the UK from 18 to 47 years [1]. As recent new medications to treat the basic defect in CFTR develop further, life expectancy is likely to continue to rise [2], but this is a progressive disease, and the complex medical management is burdensome and time consuming with frequent hospital stays.

Several factors can explain how the damaging effects of CF frequently result in a compromised nutritional status. Most people with CF have a 150–200% increased energy intake need due to increased resting energy expenditure, and lungs and breathing difficulties result in increased energy requirements [3]. Pancreatic insufficiency, affecting up to 90% of the CF population, leads to malabsorption of fat and a need to ingest more fatty food. Enzymes are needed at every meal and snack, but even with this medication, many experience a number of difficult digestive symptoms including greasy and bulky stools, frequent and difficult bowel movements, constipation, nausea, swollen painful abdomen, heart burn and loss of appetite, all of which make it difficult to meet and maintain energy requirements [4].

These symptoms impact strongly on eating, nutrition and weight status, signifying how optimal nutrition is closely linked to respiratory status. Weight and body mass index (BMI) are well established in the literature as being independent predictors of mortality in CF [5, 6]. The importance of optimal nutritional status for health and for quality of life [7] means that dietetic care, counselling and support constitute a major part of treatment and in the UK; dieticians are important members of paediatric and adult multidisciplinary teams.

From birth, there is a necessary focus on following a prescribed dietary regimen including regular weighing and monitoring of weight, and having to eat an elevated amount of calories each day. Nutritional supplements and enteral tube feeding are often needed to achieve adequate energy intake, and malnutrition remains a significant problem within CF [8]. Such an intense focus on diet, weight and exercise for disease management inevitably influences the development of eating behaviours contributing to eating difficulties in childhood [9]. While there is much emphasis on having an optimal weight to respond to the disorder, research is scarce in exploring eating behaviours and eating from psychological perspectives within this unique population.

Self-regulation, which is a primary aim for people with CF, has been explored extensively in association with eating more generically, but not within a CF population. Researchers have sought to address the problems of obesity and related diseases, to enable lower calorific consumption and aid weight loss and maintenance by regulating food consumption [10]. Findings suggest that living in an ‘obesogenic environment’ contributes significantly to the obesity issue [11, 12]. Other research has focused on the problem of multi-tasking such as watching TV while eating. Results indicate that not being attentive to the food leads to inaccurate recall of how much food is consumed, and to higher consumption [13, 14]. These cognitive and environmental factors contribute to the occurrence of what has been termed mindless eating and have been well researched in the push to tackle obesity in the general population. Mantzios et al. [15] suggested that there is potential to enhance calorific intake by utilising the findings from such research to support people with CF in enhancing their calorific intake. Egan and Mantzios [16] proposed ways in which mindful eating may assist in the self-regulation of eating as a healthier and more sustainable way of engaging with food.

Kabat-Zinn [17] described mindfulness as ‘the awareness that emerges through paying attention on purpose, in the present moment, and nonjudgmentally to the unfolding of experience moment by moment’. Similarly, mindful eating adopts such a definition of mindfulness but applies the definition to eating- and food-related experiences [18]. Mindful eating has been found to increase the pleasure in eating, while simultaneously supporting a reduction in fat and sugar consumption, grazing, impulsive food choice, compulsive eating and motivations to eat for any other reason apart from internal feelings of hunger and satiety [19–30]. The positive outcomes have also led to several programmes targeting mindful eating, such as the Mindfulness-Based Eating Awareness Training (MB-EAT) [31] and the Mindful Eating and Living (MEAL) [32], where people suffering from other chronic conditions such as type 2 diabetes [33], chronic kidney disease [34] and obesity [30, 35] benefited in improving health behaviours and outcomes.

Egan and Mantzios [16, 36] proposed how mindful eating may assist people with CF in enhancing self-regulation of eating behaviours by reducing the automatic, affect and cognitive alignments when eating, which are primarily responsible for eating and weight difficulties. This is particularly important now as new modular therapies for CF (i.e. Kaftrio, Orkambi and Symkevi) show promising results in improving the health and wellbeing of people with CF, including an increase in weight gain. Self-regulation of eating behaviours will become even more important to support people in responding to changes in weight that have never been previously experienced by many people with CF. This will necessitate unlearning a lifetime of unconventional eating behaviours that have focused on encouraging consumption of high-calorie, high-fat foods. More recently, Egan et al. [37] found that higher levels of emotional eating (i.e. eating in response to emotions) significantly predicted higher BMI in a sample of people with CF and that this relationship was moderated by both mindfulness and mindful eating. While a higher BMI is often the aim and is desirable, an increase in levels of emotional eating is not, as it may lead to other problematic eating such as binge eating and grazing.

There are several challenges with investigating eating behaviours within CF, and indeed in other chronic lifelong conditions. The focus on diet and eating as part of a treatment regimen necessitates a preoccupation with eating, and being highly attentive to eating and diet is considered good adherence alongside other health behaviours [38–40]. Interpreting research relying on self-report measures of eating behaviours can therefore be problematic as patients may answer in ways that demonstrate good adherence. There is limited evidence investigating eating behaviours within adolescent and adult CF populations, and much research focuses on the psychopathology of eating, with initial early findings suggesting that people with CF may be at risk of developing eating disorders [41]. Later studies suggested that formal eating disorders such as anorexia nervosa, bulimia nervosa and binge eating were not prevalent, but that disturbed eating attitudes and behaviours were evident. [42]

Researchers and clinicians would benefit from a greater knowledge of how people with CF experience eating. In particular, understanding the psychological and behavioural approaches to eating will help to identify the prevalence of problematic eating, and to ascertain barriers to healthy eating. Such knowledge will inform ways to better support eating behaviour to achieve the maintenance of a healthy diet and weight and to align any interventions with good psychological wellbeing.

The present study utilises semi-structured interviews to explore the everyday experiences of eating in an adult CF population, investigating whether elements of mindfulness as a barrier or facilitator of self-regulating eating behaviours.

## Methods

### Participants

All patients who were eligible to take part in the study were invited to do so on selected data collection days over 12 months. Twenty adult participants (Males = 8; *M*_age_ = 38.5, *SD* = 12.5 aged between 21 and 62 years with cystic fibrosis were recruited through Heartlands Hospital (Birmingham, UK) at scheduled appointments or during an inpatient stay. Eighteen participants were White British, one Asian British and one Indian.

Participants displayed an average BMI of 23.1 (SD = 3.1), and scores ranged from 16 to 30 ten participants reported being happy with their current weight; four reported that they would like to gain weight, two reported that they would like to lose weight, and four did not comment (see Table 1). No participants reported that they were smoking.

**Table 1:** Participant demographics

Pseudonym	Age	BMI	Ethnicity	Hospital admissions in last 12 months	Happy with current weight?
Anne	26	20	White British	2	
Ava	48	22	White British	0	Yes
Cameron	23	21	White British	0	
Charlie	37	26	White British	0	Yes, could do with losing a bit
Ellen	62	21	White British	1	Yes
Emily	45	30	White British	1	Yes
Gary	37	23	White British	5	Yes
Geraint	31	22	Indian	2	Yes
Jade	39	23	White British	4	
Janice	48	22	White British	2	Yes, but would like to gain some
Joanne	23	23	White British	0	Yes
Jules	25	16	White British	2	No, would like to gain weight
Juliet	54	29	White British	0	No, too heavy
Lena	60	24	White British	2	Yes
Mark	25	23	White British	0	Yes
Nuala	40	-	Asian British	0	
Roman	48	21	White British	1	Yes
Rory	35	25	White British	1	Suppose so
Ruby	21	-	White British	2	No
Tina	32	23	White British	0	Yes at the moment

### Semi-Structured Interview

Researchers used a semi-structured interview design as this is a flexible method that allows for use of follow up questions to gain greater insight into specific discussion points. The interview topic areas were experiences around food and eating, influences on eating behaviours (i.e. family, peers, media, health advice), food choices, engagement in food preparation, appetite and eating behaviours, social eating and additional nutritional support. Questions included: ‘What influences the way in which you eat?’ ‘How would you describe your appetite?’ ‘Do you eat when you are not hungry?’ Engagement with mindful eating practices was explored in line with previous literature suggesting how mindful eating may help in reducing barriers to eating for people with CF [16, 36, 37] and the potential implications for improving existing eating behaviours [7]. Interviews lasted for between 25 and 80 min and were conducted by two researchers in private rooms. Participation was voluntary, and pseudonyms were used to ensure confidentiality. Participants were able to withdraw from the research at any point. Ethical approval was gained from the National Research Ethics Service.

### Analysis

Interviews were conducted, recorded and transcribed by the researchers facilitating full familiarisation with the data including what was said and the way in which it was spoken [43]. The data were analysed by hand using thematic analysis following Braun and Clarke’s model [44] as this is a flexible method. The research team analysed the data using an experiential approach, initially utilising inductive, reflexive thematic analysis to create open codes. This allowed for consideration of how individuals made meaning of their eating experiences while also acknowledging that meanings and experiences are socially produced and reproduced [45]. This is particularly salient when considering the social and medical context around eating behaviours, health and weight status in people with CF.

All transcripts were fully coded line by line by the researchers in accord with the stated research aims, with each code providing a descriptive label for the quotes selected and representing something interesting or important about that section of data (e.g. the code ‘worrying about weight’ documents how many participants felt constant anxiety about their weight). The researchers presented their initial codes to one another, reflecting upon these codes, allowing revision of the initial codes based upon each researcher’s perspectives of the data. The validity of findings was supported when all researchers agreed on a common interpretation of the data. This method of triangulation demonstrates that the theories have been challenged and integrated to produce a clear understanding of eating beliefs and behaviours and mindful/mindless eating within CF. Once revision of the initial codes was complete, the researchers together generated the themes of the data by categorising the codes into meaningful groups. Through assessing how the themes supported the data and the overarching theoretical perspectives, the researchers defined what each theme was, considered which aspects of the data they had captured, and what was interesting about the themes. These collaborations helped to ensure the consistency and agreement of codes and theme generation.

## Results

Thematic analysis was employed, and five themes were constructed and developed from the data set (Table 2). The first, ‘A Spoonful of Sugar Helps the Medicine Go Down: Treatment Barriers to Eating’ explores how medical interventions for CF impact detrimentally on eating behaviours. Theme 2 ‘Watchful Weighting’ emphasises the knowledge and attentive awareness of weight (loss and gain) shown by participants. In theme 3, ‘Under Pressure: Bearing the Weight’, the effortful strategies and methods employed for gaining and maintaining weight are described. Theme 4, ‘The Proof of the Pudding is in the Eating’, explores how consumption is often focused on results (weight gain), with little or no pleasure in eating experienced by participants. In the final theme, ‘A lifetime of Cystic Fibrosis: You Live and You Learn’, the experiences of childhood and adolescent eating and how these impact in adulthood are described.

**Table 2:** Development of codes to themes

A spoonful of sugar helps the medicine go down: treatment barriers to eating	Watchful weighting	Under Pressure: bearing the weight	The proof of the pudding is in the eating	A lifetime of cystic fibrosis-you live and you learn
Impact of medical treatments on eating behaviours	Awareness and knowledge of weight/BMI	Strategies for weight gain	Eating and pleasure (food is a chore)	CF v Normative body images Weight regulation and body image
Impact of hospital stay on eating behaviours	Worrying about weight	Calories are the priority	Eating what you fancy	Skinny and CF
Hospitalisation is difficult (doesn’t get easier over time)	Weight is unstable (and is directly related to symptoms and health status)	Supplementary nutrition	No restriction of food (I eat what I like)	CF in childhood (childhood diet is focused on weight gain)
Historic CF (late diagnosis)	Weight is not the only health issue around eating behaviours	The (constant) threat of tube/peg feeding	Cooking and preparing food	CF weight and HRB in adolescence

### A Spoonful of Sugar Helps the Medicine Go Down: Treatment Barriers to Eating

Participants described several ways medical aspects of having CF impacted on the way in which they ate, the necessity of combining food and medication disrupted usual eating behaviours, sometimes it was the inconvenience of having to take medication before consuming food that prevented usual eating as Cameron describes:

Cameron (M, Age 23, BMI 21): It’s really like, you know, if I don’t have it, let’s say you’re doing something, it’s quite petty, quite silly really, but if someone said “do you want a kit-kat?” because I don't have a drink to take my creon I’ll be like naah.

At other times, it was the need to eat food with medication that impacted on usual eating. Ava describes how starting on a new medication (Kaleydeco) which needed to be taken with food that contained fat interfered with their usual eating routine and resulted in a higher consumption of convenient high fat and sugar foods leading to unwanted weight gain:

Ava (F, Age 48, BMI 22): I started to take it with my breakfast, and then, like, dinner, but it didn't work out that way, say I'd missed breakfast, I was like now I've got to have a chocolate bar, something to have this tablet.

Medication also changed the way that hunger was experienced and therefore altered eating behaviours. In Tina’s description of hunger as bizarre and weird, we understand that hunger is not usually a driving force to eat, and that feeling hungry is unusual:

Tina (F, Age 32, BMI 23): Erm, when I’m on steroids; constantly hungry then, to me it feels bizarre, ‘cause you have hunger pains, the grumbles in the belly. This is all weird to me.

While medication has an everyday impact on eating behaviours, participants also explained that hospitalisation presented additional barriers to healthy eating in both direct and indirect ways. One direct effect was described as lack of access to healthy foods and easy access to unhealthy foods:

Ellen (F, Age 62, BMI 23): Then I think I shouldn’t be doing that. On this ward it’s quite difficult, everything is snacks, “have a snack”, it’s all chocolaty, sugary. I think, I really don’t need that.

Indirectly, the very experience of being in a hospital environment, even for ‘routine’ treatments such as IV antibiotics, altered eating behaviours, perhaps through associative learning of experiencing feeling very unwell during previous visits:

Roman (M, Age 48, BMI 21): I really, really have a hard time eating hospital food now. I didn’t before, but after the last couple of times, even the smell just round dinner time puts me off food so much.

Although hospitalisation was a regular occurrence for many participants, this remained a difficult aspect of life with CF, even when it was seen as routine by relatives, carers and healthcare professionals.

Cameron (M, Aged 23, BMI 21): I think the nurses, because they see me so often, they sort of just assume it’s not a big deal. Yeah, it’s funny, ‘cause people get used to it. But for you it’s just as difficult.

Many participants talked about serious historic difficulties of managing diet and eating with CF before more recent advancements in treatments and therapies, and also of coping with undiagnosed CF before the advent of new-born screening.

Emily (F, Aged 45, BMI 30): Cause back in the day the tablets weren’t as good. I wasn’t allowed to eat chocolate, crisps, when I went to school. I had dinners, I could have the dinner, but not the pudding, and I always wanted the pudding. And then the tablets, it was about (19)87, (19)88 and then creon came along.

These early learning experiences around eating behaviours may have helped to establish and enforce a keen knowledge and awareness of weight regulation in later life, and this is explored in the next theme.

### Watchful Weighting

All participants knew what their current weight was and could give detailed information about their lowest and highest weights over long periods of time. Participants talked of weight gain as a journey and spoke about reaching a good or desired weight in ways that denoted the effort involved in such a journey.

Gary (M, Aged 37, BMI 23): Yeah but then, so yeah, I think it weren’t until, I think I remember getting to ten and a half stone. I thought okay, I feel healthy. I feel good. I know what I’m eating to maintain this. I was always eating, and I got up to just over twelve stone.

Tina (F, Aged 32, BMI, 23): Erm, my lowest weight I was five and a half stone. It took me seven years to get it, stabilise it at a healthy weight.

Monitoring weight was a constant consideration for the majority of participants, with most of the focus on gaining weight, or maintaining weight gain:

Anne: (F, Aged 26, BMI, 20) Erm, it’s always in the back of my mind, like that err, to make sure that I'm, you know, always eating, not losing weight drastically.

The effort expended to maintain weight was considerable, and sometimes simply too much, as described by Cameron below who at the time of the interview was feeling particularly despondent about his weight:

Cameron (M, Aged 23, BMI, 21): I always want to be bigger than I am, every guy has that. But the effort of having to eat, ‘cause you have to eat double the amount of calories normally, just to keep the weight I've got; let alone how much extra I'd have to eat to get more weight on. You know to build myself ooh, I just can’t, can’t be bothered with it.

The fluctuating nature of CF means that health status can be very unstable, changing from well to extremely unwell very quickly, and the need for constant vigilance over weight was even more challenging when health deteriorated. Gary spoke of a long struggle to attain a weight status with which he was happy:

Gary (M, Aged 37, BMI 23): Putting weight on is hard when you’re unwell. It’s hard, you need the treatment to help you get goodness out of it, it’s all, it’s all in a circle, you need, for me, I need, if I’m unwell, then my diet can suffer.

Almost all participants readily and easily talked of times when they had lost significant amounts of weight due to illness:

Cameron (M, Aged 23, BMI 21): There was a time a couple of years ago, I had an infection I lost so much weight, I lost like, four stone. I was like skin and bones lying in the bed.

Even when participants’ health was currently good, they talked about a constant awareness that this could change very quickly with an immediate detrimental impact on weight. Joanne discussed here how even a minor common cold can have a serious impact on weight:

Joanne (F, Aged 23, BMI 23): Recently I was really well. I’d got to the heaviest weight I’d ever been. I was really well, and then I had a cold, and then I lost ten pounds ‘cause I wasn’t eating. So it’s sort of up and down, like when I'm well, I really love food. I eat lots and I do really well. And then, if, you know, I have a chest infection or a cold, whatever, it just goes straight out the window.

One of the ways that Mark coped with this struggle to maintain weight was to focus on the present and temporary nature of the acute symptoms,

Mark (M, Aged 25, BMI 23): Erm no, if I’m unwell I struggle to eat. Erm, it takes a long time for me to force myself, even though I know once I start eating, things will settle back down. No, what I do find, if I do slow down the eating, I can drop the weight really quickly, so I have to do the constant eating.

The struggle to eat when unwell was vividly described by many participants and is more fully explored later in the theme ‘The Proof of the Pudding is in the Eating’. While weight was the predominant issue spoken about concerning eating behaviours, it was not the only consideration. Several participants spoke about trying to eat healthily, Jade talks about reducing sugar consumption and how this could improve health more holistically:

Jade (F, Aged 39, BMI 23): I don’t do diets but I think, because this one wasn’t just about weight; it was about these peaks and troughs, and I thought, well obviously with my health, energy is a big factor. So I thought, it’s not going to take away the CF, but would it help me if I at least took away the sugar highs and lows?

Several participants had CF-related diabetes, and this was an additional consideration in eating behaviours. For most of the participants, eating is primarily focused on how to prioritise CF needs, namely maintaining or gaining weight; the needs or requirements of other conditions were, unsurprisingly, seen as secondary to the primary focus of CF as Geraint explains:

Geraint (M, Aged 31, BMI 22): I haven’t tried any particular diet ‘cause what I eat is good enough anyway, so I was like maybe I should try, but the Nurse said just “look, have your insulin if you like it”, so I just went back and had what I wanted. I thought if I lost weight, it just wouldn’t be worth it.

There was sometimes a degree of confusion and perceived conflicts in dietary advice relating to a ‘good’ diet for CF and whether such advice accommodated other health concerns.

Ellen (F, Aged 62, BMI 21): Err, well the advice from the dietician, because of the Cystic Fibrosis diagnosis, and conflicts somewhat with high cholesterol. I try and steer a middle line down all of that yeah, it’s, it’s a bit of a problem to be honest yes. I need more calcium than most people because of the Cystic Fibrosis. I love to drink milk and eat cheese, and then you think, ooh cholesterol. Yeah, it’s a balance, whether I’ve found the right balance, I don’t know

The awareness and knowledge of weight status (current and past) displayed by participants was noticeably underpinned by an awareness of the strong association between health status and weight (or BMI), and the next theme explores this in more depth.

### Under Pressure: Bearing the Weight

Participants described thinking about weight maintenance or gain as a constant presence and pressure in their lives. While they were not all currently attempting to gain weight, they had all experienced times when they had needed to engage in behaviours that were specifically aimed at gaining weight. To achieve weight gain, a number of strategies were employed; some of these were directly related to the choice of food consumed and included eating ‘easy’ foods that were lighter or easier to swallow:

Lena (F, Aged 60, BMI 24): Banana sandwich. Yeah, easy to eat. I struggled with the meals, I couldn't eat the meals people put in front of me, but I could eat these other things. Err; I think a lot of it is, they’re easy to slip down.

Other strategies were not directly related to the food eaten, but were rather around the process of thinking about how and when to eat, and of preparing food, which could be deemed mindful preparation. Several people suggested eating little and often, and that eating late in the evening was useful, especially when appetite was poor:

Juliet (F, Aged 54, BMI 29): Erm, I know when I’ve not been well, that the easiest time to eat is night cause you’ve done all your treatments for that day then, and it’s the best it’s going to be. So perhaps to eat later, just have the thickened drinks earlier when you can’t face eating, and then try and have the food later when your chest is going to be the clearest it’s going to be that day and you’ve finished all your treatments.

Practical strategies related to weight gain were often focused on reducing the effort required to prepare food. For some participants, this meant disengaging from food preparation as much as possible, for example using takeaways:

Tina (F, Aged 32, BMI 23): And we literally did live on takeaways, sometimes we’d have three takeaways a day, so it was very bad, for the wallet as well as the person [laughs]. But obviously it helped me, so erm, I think more calories, err, I don’t really know, not the stress of shopping.

Food availability was a key strategic factor in supporting weight gain; when motivation to eat was low, many participants explained that it helped if food was at hand and took little effort to prepare, and convenience foods were often used to maximise calorie intake with minimal effort:

Gary (M, Aged 37, BMI 23): You need the right food in the house. Make dinner, go in the kitchen, take it out the freezer, pop pop pop, put it in the microwave. Eight minutes, I can sit down for eight minutes, watch the telly. That took twenty seconds of preparation, put it back in, take it out, put it on a plate, stir stir stir, sit on my ass. There you go. There’s seven hundred calories, with thirty seconds of hard work.

Participants talked about the need for reducing or avoiding feeling overwhelmed by food and eating when managing the pressures of maintaining or gaining weight. Sticking to the same eating behaviours and routine was helpful for some people, and limiting the variety of foods eaten both overall and at any one time was also a common strategy:

Mark (M, Aged 25, BMI 23): I tend to not stray too far from a bunch of stuff anyway, my shopping list is the same every two weeks. Bar three or four items, I go through the same foods every two to three weeks.

Lena (F, Aged 60, BMI 24): Like beans on toast, tomatoes on toast, or cream crackers and cheese. I struggled with the meals, it didn't look a lot on the plate, there was only, if I think about it visually, two items: the bread and the beans.

As well as utilising practical strategies for weight gain, participants described using behavioural and psychological tactics to increase food intake, including enlisting support from others to remind and encourage eating. Distraction was employed to help override satiety cues and to reduce thinking about eating:

Cameron (M, Aged 23, BMI 21): Oh yeah, if I’m, let’s say, watching a film at the cinema, or even just on my laptop. If I’m watching a film, I’ll always grab something, I think it’s a force of habit, rather than me being hungry. So that’s a good thing.

There was much discussion on how the need to eat reduced the enjoyment of eating, and this is more fully explored within the next theme ‘The Proof of the Pudding is in the Eating’. In terms of strategies for weight gain, participants talked about trying to focus on eating what you enjoy rather than what you think you need:

Tina (F, Aged 32, BMI 23): I think I tried to force myself to eat, and not enjoy what I was eating. When I changed it, psychologically in my own head, that’s when I started to stop eating what I think I've got to eat. ‘Cause on the board (in the hospital ward) it says to eat burgers, so I was trying to eat burgers. Which is probably why I don't like burgers anymore [laughs].

Food choices were rarely made without thought, but were more deliberated; the calorific content of food was often the predominant factor in food selection over nutritional content of food, and this focus on calories was perceived as being shared by healthcare professionals:

Anne (F, Aged 26, BMI 20): Yeah with a lot of calories in to keep my weight, I'd rather eat that sort of food to help my weight (high fat foods).

Roman: (M, Aged 48, BMI 21): I mean, I think the most important thing for them, the dieticians, is calories. They want me to have more calories.

This focus on calorie intake is understandable when considered in the context of the perceived and actual consequences of not reaching or maintaining optimum weight. The spectre of artificial feeding loomed large over many participants as an ever-present threat if they ‘failed’ in their efforts to keep themselves at a healthy weight:

Gary (M, Aged 37, BMI 23): I remember coming in and I ate and I ate and I ate. And the dietician once said to me, and it always stuck with me, if you can’t put weight on, you have to be tube fed. ‘Cause then, that to me, was, well if I can’t feed myself, if I need a machine to feed me. It made me feel angry.

The clear narrative from all participants was that everyone at some point had had to override a lack of appetite and an absence of hunger cues, and to eat when they were not hungry. This was described as forced eating by many:

Rory (M, Aged 36, BMI 25): Sometimes I will eat when I am not hungry, but it'll make me feel awful, knowing I've got to eat.

At these times, highly palatable foods were often ‘go to’ foods.

Jade (F, Aged 39, BMI 23): Yeah, and in terms of energy, because when I’m not feeling great physically, then I go for the cake or chocolate. I think it’s a short term fix, I’m trying to persuade myself to eat bananas and nuts and things like that [laughs]. It’s not the same [laughs].

Central to mindful eating practices is being aware and responding to physiological hunger and satiety cues; having to eat when not hungry was vividly described as one of the most difficult aspects of having CF with eating behaviours. How people coped was to try to consider the consumption of food as an agency of staying well:

Tina (F, Aged 32, BMI 23): I do have to be encouraged to eat, big pain in the butt, ‘cause I hate having to eat when I’m not hungry. It’s like medication, you’ve got to do it to stay well, so…..

This focus on food as fuel for life is explored in the following theme which discusses the role of pleasure and enjoyment in food and eating.

### The Proof of the Pudding is in the Eating

Everyone interviewed described particular times when eating was not pleasurable and was a chore, usually due to lack of appetite from illness and pressure to gain wait, and for some, this was a permanent, overall feeling. Cameron experiences this anhedonia even when hunger cues are felt:

Cameron (M, Aged 23, BMI 21): I’ll go down, quite hungry, and do you know when you sort of sigh, go downstairs, eat your dinner; just get nothing from it.

And for others, including Tina, not being able to use other appetite cues, such as sense of smell impacted on pleasure in eating:

Tina (F, Aged 32, BMI 23): I don’t know why I don’t enjoy eating, I think it’s probably because I’ve lost my sense of smell, so I don’t get the smell of the food.

Some people considered their overall relationship with food as different from the norm, with food being seen as an irritation rather than a source of satisfaction; Roman describes how eating is something that ‘has to be done’ and interferes with what he actually wants to be doing:

Roman (M, Aged 48, BMI 21): I’m probably not as much as a foodie as them (Friends). They seem to really like it, and live for food. I don’t necessarily enjoy food, it gets in the way. I get annoyed. Yeah, I don’t like eating. I don’t enjoy it.

When asked about their relationship with food, Ellen describes it as indifferent, or non-existent; food is eaten because it is necessary:

Ellen (F, Aged 62, BMI 21): Erm, I don’t think I really have a relationship with food. It’s necessary. I prefer to eat things that are palatable, if it was a choice between nothing and something that wasn’t palatable, I’d still eat it. Erm, but I think that’s the extent. Yeah, more of a tool really.

Only two people in this study expressed divergent views on eating and described their overall relationship with food in terms of pleasure and enjoyment.

Janice (F, Aged 48, BMI 22): Yeah # I love doing food.

Nuala (F, Aged 40, BMI ND): I would describe my relationship; I absolutely love food I love, erm, experimenting with different kinds of foods. I just love like, different flavours and tastes and experimenting. So, I do absolutely love food.

Both of these, participants discussed how the preparation and cooking of food was an important part of food enjoyment, although for Nuala, it was not her who undertook this task:

Janice (F, Aged 48, BMI 22): Love cooking, I love cooking. I like planning meals. I’ll find a recipe, ooh, I like that one, and I’m one of these I can go to the food cupboard, other’s will say ‘oh there’s nothing in’, and I’ll rumble up a dish.

Nuala (F, Aged 40, BMI ND): So, I don’t like cooking. My husband does all the cooking. He really likes cooking though. I guess food is a big part of our lives, we have people over for dinner.

A few other participants expressed that they found enjoyment in cooking at least some of the time; for others, poor health and a lack of appetite understandably reduced the motivation and ability to cook meals.

Rory (M, aged 35, BMI 25): I eat a lot of fish. I find preparing a meal a pain in the arse. Ooh, I’ve got a piece of salmon, I’ll just chuck the salmon in the microwave, zap it and eat it. I won’t like buggar around with it.

Jules (F, aged 25, BMI 16): I’m not into cooking as much, when it comes to about five o clock, oh I need to go to the supermarket. Then I’ve got to come back. Then I’ve got to eat it. Then I’ve got to wash up and then it’s time for bed. I’ve just wasted the whole night. Just don’t enjoy it anymore.

Participants worked at finding ways to induce or increase pleasure in eating, and the understanding here was that experiencing pleasure in eating at least some of the time was key to maintaining optimal nutritional wellbeing. There was much talk about ‘eating what you fancy’ in juxtaposition to eating what you think your body needs, or focusing on what or how you ‘should’ be eating:

Jules (F, aged 25, BMI 16): I’d say in recent years I’ve become more picky with my food, but I kind of, have to really fancy something to eat it. So if I get to six o clock and really fancy a pizza, I don’t really think twice about it. Yeah, just have the pizza. Yeah, who cares?

Joanne (F, aged 23, BMI 23): Most of the time it’s if I don’t fancy it I don’t eat, and then I’ll get to the point where I’m like, ok I ain’t ate for, how many hours now?

The notion that ‘I eat what I want’ was articulated a great deal in terms of there being no restriction on the kinds or amounts of food that is eaten:

Mark (M, aged 25, BMI 23): I can eat, if I want to erm, I tend to go to a restaurant it'll tend to be an all you can eat restaurant, rather than somewhere that just gives a meal, cause I can sit there and eat like, six plates.

Ruby (F, aged 21, BMI ND): Oh yeah, I’d just eat anything. I wouldn’t think twice about anything, there’s no need to.

To reduce the burden of eating, many participants engaged in significant planning and preparing and routine, and this was an important means of increasing the possibility of experiencing pleasure in eating:

Gary (M, aged 37, BMI 23): I plan everything. I know what I’m going to be eating week in, week out, pretty much to a T. Erm, routine is important.

Nuala (F, aged 40, BMI ND): To help me build up, I’ve got to plan. If I want to achieve some goals and I need extra, like, energy or something, a trip or something I’m aiming for; I need to put on some weight, I’ve got to think about it. I’ve got to get certain foods, I’ve got to be fit and healthy for that trip. I wouldn’t say I worry, I’d say, just like, I’d say more, like planning.

Eating out also served as a means of reducing the burdens associated with eating:

Charlie (M, aged 37, BMI 26): I love quality food, I love just the fact of not having to cook it, or my wife not having to cook it.

Jules (F, aged 25, BMI 16): Yeah, yeah I’d rather, I don’t know, it’s a lot easier and less caffufle [laughs]. But it does cost a lot more.

Any increase in pleasure was largely focused on the social aspects of eating out, such as the company or the environment rather than on the actual food itself:

Cameron (M, Aged 23, BMI 21): It is a treat in a sense. But I, [it’s] the social side of it, you know. I don’t get much enjoyment out of the eating. I just like being with my friends, out doing something together. I’m just going to talk, the social side and I think that’s what I enjoy more than actually having the food.

For most people, eating out was the only element of food behaviours that was considered a treat. The usual understanding of high fat and sugar foods as treats was noticeably absent in this data; one or two participants suggested that chocolate or cake might be a treat, but for the majority, food was emphatically not seen as a treat:

Cameron (M, aged 23, BMI 21): You’ve actually got to go through the bloody effort to have the treat. Take your tablets to eat it. It’s not even bloody worth it. Do you know what I mean? So you don’t have it, so I wouldn’t say it’s a treat.

Treats were seen as being something that are restricted and occasional, and foods that were understood to be usual treat foods for others were consumed widely and without restriction, and therefore did not serve as treats. The treat element of food, where it was mentioned, was confined to cost, so expensive foods were treats:

Roman (M, aged 48, BMI 21): One thing is I don’t have to restrain myself from eating anything, so it’s not like one particular food is a reward that I normally, shouldn’t indulge in.

There were many different and often difficult experiences with food and eating that contributed to a lack of pleasure in eating for most participants. The impact of these experiences over a lifetime of having CF is explored further in the next theme.

### A Lifetime of Cystic Fibrosis: You Live and You Learn

Several participants talked about CF and their eating behaviours in terms of how they differed from normative eating behaviours and what this meant for them now, and when they were younger. Childhood memories of eating were very much focused on weight gain:

Roman (M, aged 48, BMI 21): I remember the idea was just eat whatever, you know I was basically just over-indulged. ‘Cause I remember my pack lunches at school were just horrible things really. I mean, like, just, Twinkie’s and candy and other puddings. And, as far as nutrition, they weren't that great. It was largely a load of junk food.

Joanne (F, aged 23, BMI 23): When I was a kid every meal or snack I had there was like the most amount of fat there could be. If I had bacon and tomato, there’d be butter.

The difficulties of managing food and eating during childhood were clear, and in Cameron’s account, we see how their parents engaged in detrimental behaviour management strategies to try to reinforce eating:

Cameron (M, Aged 23, BMI 21): Horrible. I mean, I remember times when my Mom used to make me sit in a room for hours to eat my dinner ‘cause I didn't want to do it. I didn’t want to eat it. I don’t know if it was because I didn’t like it because I felt ill. Sometimes if you take creon too much or too less it can give you stomach problems.

As participants became more independent, adolescence was typically a time where healthy eating was not a primary focus, and risky health behaviours became a part of life for some:

Charlie (M, aged 37, BMI 26): I did have a wild sort of period between sixteen and nineteen where I was off the scales. I was crazy, crazy, crazy. Did a lot of pills and drugs. Yeah, that really fucked with my weight. You know, I was a teenager going through; well, teenagers go through a lot. Who am I? You know, fitting in and everything else; and then I’d got Cystic Fibrosis on top.

For many, attitudes and behaviours towards food changed and became more health-focused with age; participants described having a growing awareness and acceptance that what was healthy for ‘someone with CF’ was different from the norm with all the contradictions this posed:

Anne (F, aged 26, BMI 20): Some people may say pass a comment “oh you don’t need to lose weight”. It’s, for a CF patient, it’s quite difficult when you’re like, eating trying to put weight on.

Being different was discussed a great deal; the ability to avoid being obviously identifiable as someone with CF was important for self-identity, and being skinny was perceived as a ‘give away’ of CF.

Gary (M, aged 37, BMI 23): It weren’t until I put weight on that I thought, I look better, I look healthier. So I suppose, when people look at me, they will think “he doesn’t look any different from anyone else”.

Many participants from a young age lived with the expectation that life would be short, and they talked about how this impacted strongly on health experiences and behaviours:

Ava (F, aged 48, BMI 20): I remember being told I wouldn’t last past fifteen, so here we are [laughs]. I think I was probably; I was probably, nine or ten. I suppose I knew, you know, I knew I wouldn’t get as old as everyone else. But I know coming towards fourteen, fifteen, I was thinking well I’m here now.

In adulthood, the benefits of accepting an illness identity in terms of reduced risk-taking behaviour and improved adherence, including eating behaviours, were clearly expressed.

Overall, participants’ narratives repeatedly emphasised that food and eating were often a time-consuming and effortful activity that at times were the cause of much anxiety and distress. There was clear agreement that eating rarely happened without thought and planning, and all participants described strategies that they utilised to enable and support self-regulation of eating. The five themes identified in this work, from the focus on weight and calories, to the strategies for eating well for CF, will be explored and discussed with a focus on relevant literature in self-regulation, mindfulness and mindful eating.

## Discussion

All participants talked readily and easily about experiencing problems with eating, and these were attributed to both symptoms of, and treatments for, CF. Having to take the medication with food, hospitalisation and childhood experiences of food and eating were all seen to impact negatively on current eating experiences and behaviours. It is understandable and noted in the literature that an intense focus on diet for disease management inevitably impacts eating behaviours [46].

Overall, participants’ narratives demonstrated extensive knowledge of food and nutrition and showed that food and eating occupied a large part of life for people with CF. This encompassed both behaviours and cognitions, including thinking about eating, working at overcoming poor appetite and trying to eat healthily for CF, and this is consistent with previous research [8, 37]. A great deal of thought and effort was required, far more than people wanted to be thinking about eating, and much of the talk around food and eating behaviours were concerned with trying to make eating more manageable and less difficult.

Participants were highly aware of their current weight status and also of their highest and lowest weights. Vigilance around weight was strongly evident, and this is reinforced as regular weighing and discussion of diet from an essential part of quality care. Eating behaviours are therefore very much in the clinical domain. Eating for CF rather than general health or other comorbidities such as diabetes was the primary consideration in making decisions about what to eat. High fat and high sugar foods were important in enabling self-regulation when high calorie rather than high nutrition food was the priority. This focus is understandable given the strong association between nutritional and respiratory status [5, 6].

Eating was also focused on physical benefits for CF rather than psychological gains of satisfaction or pleasure; indeed, these elements were largely non-existent within this research. Even when weight was stable and eating was not particularly difficult, it was rarely described as pleasurable and was often a source of anxiety. Enteral tube feeding was unanimously perceived as an ever-present threat that demonstrated a failure to self-regulate eating and illustrated the lack of personal control and choice over eating and an inability to keep oneself alive. These findings are aligned to the evidenced reluctance to consent to tube feeding even when malnutrition is life-threatening [8].

Seeking a degree of normality and pleasure in eating behaviours was evident when participants talked about going out to eat. The treat element of eating out was not focused on food, but rather that it was an infrequent, expensive activity. This is similar to previous research in the general population [48] but the treat and the pleasure elements were even less associated with food factors such as wider choice and not needing to prepare food. Encouraging a focus on the treat and the social aspect of eating out may make eating a more joyful and mindful experience with attentive presence, which are aspects of mindful eating. It may also support psychological wellbeing as it brings a sense of normality and temporarily diminishes the “illness identity” [38, 39].

Participants frequently used the phrase ‘Eating what I want’ as a way of describing being able to eat unlimited high-fat and high-sugar foods. This phrase is an interesting choice when considered alongside the contradictory evidence from participants where they describe having to eat when they do not want to eat, to eat specific foods and to avoid certain foods. In fact, they frequently do not eat what they want. They also talked about ‘eating what you fancy’ as making a positive food choice, and this was perhaps more closely aligned to how we might usually understand eating what you want.

Hunger and satiety, usual drivers of eating behaviour, and fundamentals of mindful eating, were not strongly cited as influencing eating. The attentiveness of the present eating experience alone may not be useful for everyone with CF as this creates a focus on eating, which may increase the perception of eating as being a treatment. It is specific elements of mindful eating rather than attentive or focused eating that may be beneficial to people with CF: non-judgment, acceptance and self-compassion may help with a reduction in emotional eating and in increasing pleasure in eating, even if this is momentary or occasional. Practising mindfulness entails experiencing the present moment, whether it is pleasant or unpleasant, with qualities of awareness, non-judgement, acceptance and compassion. When self-regulation of eating is difficult and time-consuming, understanding and practising these elements may be helpful for both physical and psychological health [16, 22, 25, 36, 37, 47, 48].

Eating behaviours in people with CF leading to being overweight and obese is a growing issue [50]. This is due to improved clinical treatments including earlier diagnosis of CF and pancreatic insufficiency and enhanced medical therapies including CFTR-targeted therapies. Furthermore, dietary and eating behaviour advice to consume a higher fat diet than non-CF people that has been long endorsed may not be as consistently relevant as intake of high-fat foods has increased in the general population. The changing nature of CF in both symptoms and health status necessitates that support for eating offers flexible and easily implemented strategies. Interventions to support eating experiences and behaviours need to be adaptive to fluctuations in health status which alter nutritional needs, requiring a change in how a person regulates their eating according to the current clinical assessment of the individual.

The availability of modulator therapies for people with CF has significantly changed CF care and treatment, with reports of dramatic improvements in physical health for some people. While these improvements are long awaited and welcomed, they bring new challenges in supporting the psychological wellbeing of people through this period of adjustment and beyond [51]. Eating attitudes and behaviours are a key element of both physical and psychological wellbeing and do therefore require continuous observation, investigation and intervening. The present research has led to the development of mindful eating interventions specific to cystic fibrosis that support fluctuating health status, help to manage comorbid health conditions and enhance psychological wellbeing. Healthcare professional support for the utility of the interventions has been established, and initial evaluations are that they will compliment and extend current interventions.

## Data Availability

The datasets generated during and/or analysed during the current study are available from the corresponding author on reasonable request.

## Declarations

The study was approved by NRES Committee East Midlands—Nottingham 2 and the Ethical Review Board of the University, and was in accordance with the ethical standards of the institutional and/or national research committee, and with the 1964 Helsinki Declaration and its later amendments. This article does not contain any studies with animals.
